# Carbamazepine overdose after exposure to simethicone: a case report

**DOI:** 10.1186/1752-1947-2-242

**Published:** 2008-07-24

**Authors:** Ozlem Guneysel, Ozge Onur, Arzu Denizbasi, Murat Saritemur

**Affiliations:** 1 Marmara University, School of Medicine, Department of Emergency Medicine, Istanbul, Turkey

## Abstract

**Introduction:**

Carbamazepine is an anticonvulsant drug and is also used as a treatment for patients with manic-depressive illness, post-herpetic neuralgia or phantom limb pain. The drug itself has many drug interactions. Simethicone is an antifoaming agent and is reported to be an inert material with no known drug interaction with carbamazepine.

**Case presentation:**

We present a case of a patient who was routinely using carbamazepine 400 mg three times per day and levetiracetam 500 mg twice daily, and experienced carbamazepine overdose after exposure to simethicone. After cessation of simethicone therapy normal drug levels of carbamazepine were obtained again with the standard dose of the drug. The mechanism of interaction is unknown but the risk of overdose should be considered when prescribing simethicone to a patient who is using carbamazepine.

**Conclusion:**

Simethicone and carbamazepine, when taken together, may be a cause of carbamazepine toxicity. The risk of carbamazepine overdose should be considered when prescribing simethicone to a patient who is using carbamazepine.

## Introduction

Carbamazepine (CBZ) is an anticonvulsant drug which received approval for use as an anti-epileptic agent in the United States in 1974. It is also used as a treatment for patients with manic-depressive illness, post-herpetic neuralgia or phantom limb pain. Therapeutic plasma concentration is 4 to 12 mg/l. It is approximately 75% to 80% protein-bound. CBZ is oxidized by hepatic microsomal enzymes to produce its active metabolite, CBZ 10, 11-epoxide. In terms of drug interactions, CBZ induces the metabolism of other anticonvulsant drugs. Inhibitors of hepatic microsomal enzymes such as erythromycin, clarithromycin and cimetidine increase CBZ levels [1]. Cytochrome P450 3A4 (CYP3A4) inhibitors inhibit CBZ metabolism and can thus increase plasma levels; CYP3A4 inducers can increase the rate of its metabolism.

Simethicone is an antifoaming agent that acts by altering the surface tension of mucus-entrapped gas bubbles in the digestive tract, allowing them to coalesce and disperse [2]. It is not absorbed, and is excreted unchanged in the feces [3]. There is no known interaction of this drug with CBZ.

We present the case of a patient in whom simethicone is the probable cause of CBZ toxicity. After cessation of simethicone therapy normal drug levels of CBZ were obtained again with standard dose of the drug.

## Case presentation

A 45-year-old man with a medical history of epilepsy presented to the emergency room with complaints of vertigo, gait disturbance, dizziness, slurred speech and diplopia. He had been using CBZ (Karazepin^®^) 400 mg three times per day for 4 years and levetiracetam (Keppra^®^) 500 mg twice daily for 1 year. No other toxin, alcohol, herbal products or drugs were reported except for a history of 2 days simethicone (Metsil^®^) usage for abdominal distention. He had a blood pressure of 115/70 mmHg, a pulse rate of 88 beats per minute and a respiratory rate of 18 breaths per minute. The patient had a normal mental status and was able to give a reliable history. There were no signs of dehydration. He reported no history of vomiting or diarrhea. He denied trauma, extra drug dosage or suicidal attempt. In his neurological examination, the pathological findings were bilateral nystagmus, dysarthria, diplopia and ataxic walking. He had a serum CBZ level reported as 10.5 μg/ml 2 days earlier (blood sampled 10 to 11 hours after the last dose of CBZ; normal reference range 4 to 11 μg/ml), confirmed in the neurology outpatient clinic.

The initial laboratory findings were as follows: hematocrit, 41.5; prothrombin time, 12.3 seconds (reference range 11 to 13 seconds); international normalized ratio, 1.02; creatinine, 0.97 mg/dl (reference range 0.5 to 1.10 mg/dl); glucose, 102 mg/dl (reference range 70 to 110 mg/dl); serum alanine aminotransferase, 16 U/L (reference range 10 to 37 U/l); aspartate aminotransferase, 17 U/l (reference range 10 to 40 U/l); alkaline phosphatase, 238 U/l (reference range 0 to 270 U/l); γ-glutamyltransferase, 46 U/l (reference range 7 to 49 U/l); total bilirubin, 0.54 mg/dl (reference range 0.2 to 1.0 mg/dl); and CBZ serum level, 34.2 μg/ml (blood taken 8 to 9 hours after the last dose of CBZ).

Cranial computed tomography scan was normal. Confirming the simethicone levels would have been helpful in confirming the patient's report, but this test was not available.

The patient was taken to the neurology in-patient unit with a diagnosis of CBZ intoxication. CBZ was withdrawn. Treatment for intoxication comprised intravenous hydration and cardiac monitoring. After 36 hours his serum CBZ level had normalized (17.6 μg/ml at 24 hours; 11.4 μg/ml at 36 hours) and neurological exam was intact. In a follow-up visit the patient was warned about simethicone use, and there have been no further problems in the following 6 months.

## Discussion

CBZ is one of the most commonly prescribed drugs for the prevention of partial seizures as well as for treatment of generalized tonic-clonic seizures and trigeminal neuralgia [4]. CBZ interacts with a number of drugs other than anticonvulsants and there are a number of mechanisms involved. The absorption of oral CBZ is slow, erratic and unpredictable. Peak plasma concentrations generally occur 4 to 8 hours after ingestion, but may require up to 26 hours to peak. It is rapidly distributed into the body and has about 75% to 78% protein binding. CBZ is metabolized in the liver by the cytochrome P450 system and undergoes almost complete biotransformation to several metabolites. The most important interactions affecting the characteristics of CBZ are those resulting in the induction of its metabolism. Clinically, a variety of drug interactions between CBZ and co-administered drugs have been reported. The actions of most drugs that affect CYP3A4 by inhibition or induction manifest as drug interactions with CBZ [5,6]. However, there has been no demonstration of simethicone and CBZ interaction until now.

Simethicone has been used as an adjunct in the treatment of various clinical conditions in which gas retention may be a problem, including dyspepsia, infant colic, peptic ulcer and irritable colon. It also appears to be helpful as an adjunct to various procedures such as colonoscopy and bowel radiography [7]. It is a mixture of liquid dimethylpolysiloxanes which have antifoaming activity. It acts in the stomach and intestines by altering the surface tension of gas and mucus bubbles, enabling them to coalesce. It is reported as physiologically inert, and no toxic effects are reported on ingestion [8].

As it is widely available, CBZ is a drug commonly involved in accidental and intentional overdoses. The American Association of Poison Control Centers reported a total number of 18,201 CBZ overdoses from 1999 to 2001, leading to 18 deaths [9]. Acute CBZ toxicity presents with cardiac, respiratory and neurological effects. Neurological signs include loss of consciousness, seizures, ataxia, choreoathetosis, myoclonus, motor restlessness, mydriasis and nystagmus [10]. Most of these signs were positive in our patient.

We have presented a case of CBZ toxicity due to simultaneous intake of simethicone. The Naranjo adverse drug reaction probability scale was used as an objective measure of causality; a score of 7 was found [11]. Based on a score of 7 on the Naranjo adverse drug reaction probability scale, simethicone was the probable cause of CBZ toxicity in this patient. Both drugs are in wide use, but to date there have been no studies examining the effects of simethicone on the pharmacokinetics of CBZ. As simethicone is an inert material, we could not explain the mechanism of action. This interaction may be caused by extrahepatic enzymatic processes. There are some reports regarding absorption processes and CBZ therapeutic levels. It was confirmed that simultaneous oral administration of TJ-9 (Sho-saiko-to extract powder) with CBZ to rats decreased gastrointestinal absorption of CBZ, without affecting the metabolism of CBZ [12]. In another study it was shown that concomitant administration of Coca-Cola (an acidic beverage) enhanced the rate and extent of absorption of CBZ [13]. An absorption effect may therefore be responsible for CBZ toxicity in a patient taking simethicone and CBZ together.

In a patient with CBZ toxicity, possible causes such as multiple drug ingestions, beverages, herbal products, drug overdoses and liver function abnormalities should be considered. Our patient had been taking CBZ for 4 years; during this period no adverse events were reported until simethicone usage. This patient's follow-up was excellent.

## Conclusion

Simethicone and CBZ, when taken together, may be a cause of CBZ toxicity. The risk of CBZ overdose should be considered when prescribing simethicone to a patient who is using CBZ.

## Abbreviations

CBZ: carbamazepine; CYP3A4: Cytochrome P450 3A4.

## Competing interests

The authors declare that they have no competing interests.

## Authors' contributions

OG and OO were involved in the literature search, writing and conception of the report. AD and MS conceived of and gave final approval to the report. All authors read and approved the final manuscript.

## Consent

Written informed consent was obtained from the patient for publication of this case report and accompanying images. A copy of the written consent is available for review by the Editor-in-Chief of this journal.
